# Microbe Profile: Plasmodiophora brassicae, the gall-forming protist behind clubroot disease

**DOI:** 10.1099/mic.0.001774

**Published:** 2026-09-18

**Authors:** Marcos Calcaterra, Jose Luis Valdez-López, Muhammad Asim Javed, Edel Pérez-López

**Affiliations:** 1 Département de phytologie, Faculté des sciences de l'agriculture et de l'alimentation, Université Laval, Québec City, Québec, Canada; 2 Centre de recherche et d'innovation sur les végétaux, Université Laval, Québec City, Québec, Canada; 3 Institute de Biologie Intégrative et des Systèmes, Université Laval, Québec City, Québec, Canada; 4 L’Institute EDS, Université Laval, Québec City, Québec, Canada

**Keywords:** ankyrin-repeat effectors, clubroot, effectors, hormones, plasmodiophorids

## Abstract

*Plasmodiophora brassicae* is a soil-borne intracellular obligate biotrophic protist and the causal agent of clubroot disease in Brassicaceae. Phylogenetically placed within the Rhizaria supergroup, it exhibits a complex two-stage life cycle leading to the formation of club-shaped galls, driven by phytohormone and effector-mediated host reprogramming. A recently obtained telomere-to-telomere genome assembly of the Canadian pathotype *Pb3A* revealed a compact genome structure encoding a complex secretome. Despite its agronomic relevance, key aspects of its biology, including the existence of sexual reproduction, host specificity determinants and effector biology, remain poorly understood, due to its unculturable nature and limited genomic resources.

## Graphical abstract

*Plasmodiophora brassicae* symptoms and life cycle. Club-shaped *Brassica napus* root galls (right) and life-cycle model adapted from Javed *et al*. [1] (left), showing primary and secondary infection, zoospore development and resting-spore formation.

## Taxonomy

Domain: Eukaryota; Supergroup: Rhizaria; Class: Phytomyxea; Order: Plasmodiophorales; Family: Plasmodiophoraceae; Genus: *Plasmodiophora*; Species: *Plasmodiophora brassicae* (Woronin, 1877).

## Properties

*Plasmodiophora brassicae* is a soil-borne obligate biotrophic protist that colonizes Brassicaceae roots [1]. Like fungi, *P. brassicae* forms chitin-containing resting spores, but features such as cruciform nuclear division, biflagellate secondary zoospores, multinucleate plasmodia, uninucleate resting spores and lack of filamentous growth distinguish *P. brassicae* from true fungi. Its intracellular lifestyle makes its experimental manipulation challenging [1]. *P. brassicae* characteristically induces nutrient-sink galls through stimulation of meristematic activity and sugar reallocation to infection sites [2]. However, gall formation appears to enhance rather than determine pathogen fitness, as *P. brassicae* can complete its life cycle in roots with minimal gall development, although spore production is substantially reduced [1, 2].

## Genome

The *P. brassicae* genome is compact due to low intergenic regions and transposable elements relative to most eukaryotic pathogens, reflecting its obligate intracellular lifestyle [3, 4]. Recent telomere-to-telomere (T2T) assembly of the Canadian pathotype *Pb3A* revealed a ~25.3 Mbp genome distributed across 20 chromosomes [4]. This T2T assembly has a 59.4 mol% G+C content, nearly 3 Mbp identified as repeat regions comprising mainly interspersed repeats (Gypsy, Copia, SINE and LINE), and ~10,500 protein-coding genes. It represents the first complete genome assembly for any member of Rhizaria, and the reference genome for *P. brassicae* available in the NCBI database (GCA_036867785.1) [4].

The *P. brassicae* genome is enriched in genes associated with host interaction, including secreted effectors and expanded protein families such as ankyrin-repeat proteins, which are highly expressed during infection and likely contribute to host manipulation [5]. Comparative analyses reveal a markedly reduced genome relative to free-living Rhizaria, consistent with the reductive evolution expected in obligate intracellular biotrophs. Despite this, the pathogen retains a diverse repertoire of metabolic and virulence-associated genes, highlighting a streamlined but highly specialized lifestyle. Notably, most predicted genes remain functionally uncharacterized, reflecting both the limited representation of plasmodiophorids in functional databases and their deep evolutionary divergence from other well-studied eukaryotic lineages [1, 4–6].

## Phylogeny

*P. brassicae* belongs to the supergroup Rhizaria, within the class Phytomyxea and order Plasmodiophorales. Phytomyxea are obligate biotrophic parasites found in freshwater, marine and soil environments, infecting plants, algae, diatoms and stramenopiles. Phytomyxea are characterized by complex life cycles, intracellular development and host specialization. Within Phytomyxea, *P. brassicae* belongs to plasmodiophorids, a group of highly specialized plant-infecting microbes. In rare cases, plasmodiophorids infect alternative hosts but are unable to complete their life cycle, and infection is restricted to individual cells [1, 7].

## Key features and discoveries

Recent advances in effector biology have revealed that *P. brassicae* employs a remarkably sophisticated repertoire of host-manipulation strategies [8]. One of the clearest examples is the targeting of plant hormonal pathways [9]. PbBSMT, a SABATH-type methyltransferase, converts salicylic acid into methyl salicylate, weakening both local and systemic immune responses, recently also shown to methylate and neutralize the immune signalling compound N-hydroxy-pipecolic acid [10]. Additional effectors interfere with jasmonic acid and ethylene-associated pathways, demonstrating that hormonal manipulation is a central component of pathogen virulence rather than a secondary consequence of infection [9].

A second major discovery is the diversity of mechanisms used to suppress plant immunity. Secreted E3 ubiquitin ligases promote degradation of immune-related proteins such as RD21A, cystatin-like effectors inhibit host proteases and CBM18-containing proteins bind chitin and suppress chitin-triggered immune responses [1, 8, 10]. Recent work has also demonstrated that *P. brassicae* manipulates host metabolism and gene expression directly. PBZF1 targets SnRK1.1, a central regulator of plant energy homeostasis, while PbZFE1 functions as a transcription factor-like effector capable of directly modulating host gene expression [1, 8, 10]. These findings provide a mechanistic explanation for how the pathogen redirects host resources towards gall development and resting spore production.

Beyond the characterization of individual effectors, comparative analyses have uncovered broader evolutionary patterns. Several *P. brassicae* proteins share structural similarities with virulence factors described in bacteria, fungi, oomycetes and other protists, despite limited sequence conservation [6]. Particularly intriguing is the identification of glycoside hydrolase-like and other conserved structural folds associated with organisms capable of inducing galls, tumours or other similar extended phenotypes in plants. These observations suggest that evolutionarily distant microbes may repeatedly converge on similar molecular solutions to manipulate host development.

At the same time, *P. brassicae* possesses molecular features that appear distinctive among plant pathogens. The expansion of ankyrin-repeat proteins and kinase-domain-containing effectors, together with other lineage-specific secreted proteins, points to the existence of plasmodiophorid-specific mechanisms of host manipulation.

The repeated emergence of *P. brassicae* isolates capable of overcoming genetic resistance has become a defining feature of the clubroot pathosystem [1, 8]. This has stimulated the development of pathotyping schemes, molecular diagnostic tools and genomic resources to monitor pathogen populations and track virulence evolution. Although the precise molecular determinants underlying resistance breakdown remain largely unresolved, the integration of pathogen genomics, effector biology and molecular surveillance is beginning to provide a framework for understanding how virulent isolates emerge, spread and adapt to resistant hosts [1, 8].

Collectively, these discoveries have transformed our understanding of clubroot pathogenesis and positioned *P. brassicae* as a valuable model for studying the evolution of host reprogramming, gall formation and obligate biotrophy. Emerging technologies such as single-cell and spatial transcriptomics are expected to further accelerate discoveries by enabling the study of pathogen development, effector biology and host responses at cellular resolution, providing a more precise understanding of the molecular events underlying clubroot disease.

## Open questions

1. How do *P. brassicae* effectors reprogram host development and immunity?
2. How did genome reduction shape the evolution of an obligate intracellular lifestyle?
3. What evolutionary forces drive the emergence of new virulent pathotypes?
4. Why is *P. brassicae* almost exclusively associated with Brassicaceae hosts?
5. Is *P. brassicae* evolving towards a more symbiotic relationship with its hosts?

## Abbreviations

T2T: telomere-to-telomere
